# Ursolic and Oleanolic Acids Induce Mitophagy in A549 Human Lung Cancer Cells

**DOI:** 10.3390/molecules24193444

**Published:** 2019-09-23

**Authors:** Nayeli Shantal Castrejón-Jiménez, Kahiry Leyva-Paredes, Shantal Lizbeth Baltierra-Uribe, Juan Castillo-Cruz, Marcia Campillo-Navarro, Alma Delia Hernández-Pérez, Alexandra Berenice Luna-Angulo, Rommel Chacón-Salinas, Ramón Mauricio Coral-Vázquez, Iris Estrada-García, Luvia Enid Sánchez-Torres, Carlos Torres-Torres, Blanca Estela García-Pérez

**Affiliations:** 1 Departamento de Microbiología, Escuela Nacional de Ciencias Biológicas, Instituto Politécnico Nacional, Prolongación de Carpio y Plan de Ayala s/n, Ciudad de México 11340, Mexico; naye_nice85@hotmail.com (N.S.C.-J.); kahiryley@hotmail.com (K.L.-P.); shantal_baliz@hotmail.com (S.L.B.-U.); juancast0508@gmail.com (J.C.-C.); 2 Área Académica de Medicina Veterinaria y Zootecnia, Instituto de Ciencias Agropecuarias-Universidad Autónoma del Estado de Hidalgo, Av. Universidad km. 1. Exhacienda de Aquetzalpa A.P. 32, Tulancingo 43600, Hidalgo, Mexico; 3 Departamento de Inmunología, Escuela Nacional de Ciencias Biológicas, Instituto Politécnico Nacional, Prolongación de Carpio y Plan de Ayala s/n, Ciudad de México 11340, Mexico; marcy265@hotmail.com (M.C.-N.); rommelchacons@yahoo.com.mx (R.C.-S.); iestrada5@hotmail.com (I.E.-G.); luviasanchez@hotmail.com (L.E.S.-T.); 4 Laboratorio de Inmunología Integrativa, Instituto Nacional de Enfermedades Respiratorias Ismael Cosio Villegas, Calz. de Tlalpan 4502, Belisario Domínguez Secc. 16, Ciudad de México 14080, Mexico; 5 Departamento de Anatomía Patológica, Instituto Nacional de Rehabilitación, México-Xochimilco No. 289. Arenal de Guadalupe, Ciudad de México 14389, Mexico; almadeliahp@gmail.com; 6 Departamento de Neurociencias, Instituto Nacional de Rehabilitación, México-Xochimilco No. 289, Arenal de Guadalupe, Ciudad de México 14389, Mexico; lunangulo@gmail.com; 7 Unidad de Desarrollo e Investigación en Bioprocesos (UDIBI), Escuela Nacional de Ciencias Biológicas, Instituto Politécnico Nacional, Prolongación de Carpio y Plan de Ayala s/n, Ciudad de México 11340, Mexico; 8 Sección de Estudios de Posgrado e Investigación, Escuela Superior de Medicina, Instituto Politécnico Nacional, Salvador Díaz Mirón esq. Plan de San Luis S/N, Miguel Hidalgo, Casco de Santo Tomas, Ciudad de México 11340, Mexico; rmcoralv@gmail.com; 9 Subdirección de Enseñanza e Investigación, División de Investigación Biomédica, Centro Médico Nacional 20 de Noviembre, Instituto de Seguridad y Servicios Sociales de los Trabajadores del Estado, Félix Cuevas 540, Col del Valle Sur, Ciudad de México 03100, Mexico; 10 Sección de Estudios de Posgrado e Investigación, Escuela Superior de Ingeniería Mecánica y Eléctrica Unidad Zacatenco, Instituto Politécnico Nacional, Gustavo A. Madero, Ciudad de México 07738, Mexico; ctorrest@ipn.mx

**Keywords:** ursolic acid, oleanolic acid, mitophagy, A549 human lung cancer cells, reactive oxygen species, PINK1/Parkin

## Abstract

Ursolic and oleanolic acids are natural isomeric triterpenes known for their anticancer activity. Here, we investigated the effect of triterpenes on the viability of A549 human lung cancer cells and the role of autophagy in their activity. The induction of autophagy, the mitochondrial changes and signaling pathway stimulated by triterpenes were systematically explored by confocal microscopy and western blotting. Ursolic and oleanolic acids induce autophagy in A549 cells. Ursolic acid activates AKT/mTOR pathways and oleanolic acid triggers a pathway independent on AKT. Both acids promote many mitochondrial changes, suggesting that mitochondria are targets of autophagy in a process known as mitophagy. The PINK1/Parkin axis is a pathway usually associated with mitophagy, however, the mitophagy induced by ursolic or oleanolic acid is just dependent on PINK1. Moreover, both acids induce an ROS production. The blockage of autophagy with wortmannin is responsible for a decrease of mitochondrial membrane potential (Δψ) and cell death. The wortmannin treatment causes an over-increase of p62 and Nrf2 proteins promote a detoxifying effect to rescue cells from the death conducted by ROS. In conclusion, the mitophagy and p62 protein play an important function as a survival mechanism in A549 cells and could be target to therapeutic control.

## 1. Introduction

Ursolic and oleanolic acids are ubiquitous compounds widely distributed in plants and fruits as a result of a fascinating secondary metabolism. The compounds produced by their typical primary metabolism are necessary for growth and development, while the compounds emerging from secondary metabolism have other functions that are still being elucidated. Both acids are synthetized from isoprene and have 30 carbon atoms organized in five pentacyclic rings; based on this characteristic structure, both acids are classified as triterpenes [1].

Different studies have suggested that ursolic and oleanolic acids have anti-cancer activity [2], and regarding that molecular organization of these structural isomers can show important physical and chemical actions, in this direction, further investigation seems to be an attractive topic of research. Ursolic acid has been evaluated against different models of cancer, cervical [3], prostate [4,5], and lung [6]; among others. The anticancer activity of oleanolic acid has been described against hepatocellular carcinoma cells [7] and different lung cancer cells, such as NSCLC, A549, and H460 [8]; and it has been also used in breast cancer and prostate cancer cells models [9]. Some studies have compared the biological activity of both acids. The variances in biological activity might be due to structural differences, mainly a methyl group in the E ring of oleanolic acid [1].

Autophagy is a self-degradative pathway depending on the lysosome, which is involved in the capture and elimination of unnecessary intracellular components of the cells (protein aggregates, damaged organelles, intracellular pathogens, etc.). Autophagy enables the maintenance of cellular homeostasis and regeneration of metabolic precursors. Autophagy can be a selective and non-selective mechanism. Various types of selective autophagy can be distinguished by considering the cargo that is captured and degraded; including mitochondria (mitophagy), lipid droplets (lipophagy), and pathogens (xenophagy); among others [10]. Selective autophagy implies the recruitment of adaptor proteins, such as p62/SQSTM1, NDP52, optineurin, NBR1, etc.; non-selective autophagy is induced during starvation [11].

The role of autophagy in cancer can be viewed as a controversial process. Some evidences indicate that autophagy prevents cancer development [12] but on the other hand it has been reported that autophagy has a protective role for cancer cells [13]. For that, autophagy has been targeted for stimulation and inhibition for therapeutic approaches but it is necessary a clear understanding about the context of autophagy in tumor progression. A recent review report elegantly explains the context-dependent role of autophagy on cancer [14]. The modulation of autophagy, or the molecules involved in the signaling pathways that trigger autophagy, could be targets for investigating and discovering new anticancer agents [15,16]. In that perspective, it has been reported that ursolic and oleanolic acids stimulate autophagy in cancer cells [3,17,18,19]. The mechanism by which the triterpenes induce autophagy has not been determined. Some studies have reported that ursolic acid induces autophagy by the modulation of the PI3K/AKT pathway [4] or by JNK activation [19,20]. Autophagy induced by oleanolic acid has been associated with JNK and mTOR pathways [17]. Both triterpenes can affect mitochondria activity [21] by inducing ROS production [22,23,24,25], which is a well-known trigger of mitophagy in some models; however, little is known about the relation between mitochondrial changes and ursolic or oleanolic acid-induced autophagy. In view of these considerations, the aim of the present study was to evaluate the response of the A549 human lung cancer cells taking into account an in vitro model of lung cancer together to the stimulation by ursolic and oleanolic acids. Our results in this important topic allowed us to establish the role of fundamental mechanisms involved in particular autophagy phenomena, which can be considered as a base for future research about autophagy in the cells survival.

## 2. Results

### 2.1. Ursolic and Oleanolic Acids Decrease the Viability of A549 Human Lung Cancer Cells

**Figure 1:**
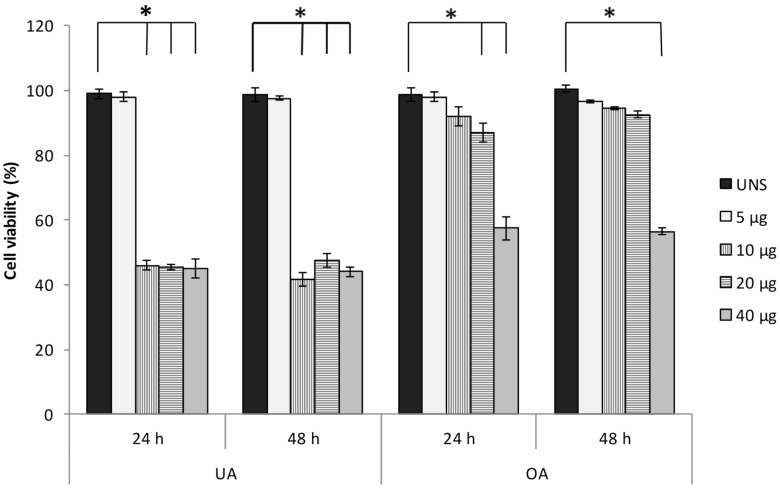
The effect of ursolic and oleanolic acids on A549 human lung cancer cells viability. A549 cell monolayers were stimulated with several concentrations of ursolic acid (UA) or oleanolic acid (OA) (5, 10, 20, and 40 µg/mL) and trypan blue assay was performed. The results are presented as the means and standard deviation of triplicate experiments * statistically significant differences (*p* < 0.05) when the stimulated cells were compared with untreated cells (UNS).

To verify the effect of ursolic and oleanolic acids on the viability of A549 cells, trypan blue assay was performed. For both acids, the lower concentration analyzed (5 µg/mL) does not cause cytotoxic effect. However, at 24 and 48 h post-stimuli, the upper concentrations (10, 20, and 40 µg/mL) of ursolic acid induced remarkable decrease of cell viability (approximate 50%). On the other hand, oleanolic acid induced a doses-response effect at 24 h and 48 h post-treatment, and only 40 µg/mL oleanolic acid caused a notable decline in cell viability (56%) (Figure 1). These results indicate that ursolic acid presents more cytotoxicity than oleanolic acid on A549 cells. Based on these results, the following experiments were carried out with 10 µg/mL ursolic acid or 20 µg/mL oleanolic acid.

### 2.2. Ursolic and Oleanolic Acids Induce Autophagy in A549 Human Lung Cancer Cells

**Figure 2:**
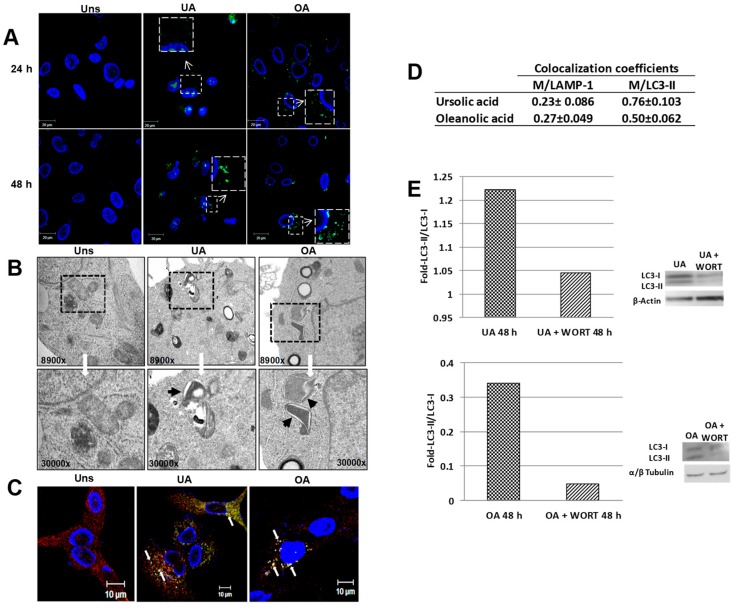
Ursolic and oleanolic acids induced autophagy in A549 human lung cancer cells. (**A**) A549 cells unstimulated (Uns) or stimulated with 10 µg/mL ursolic acid (UA) or 20 µg/mL oleanolic acid (OA) for 2, 6, 24, and 48 h were fixed with 4% paraformaldehyde and washed with PBS. Subsequently, the cells were incubated with an anti-LC3A/B antibody at 4 °C overnight and an anti-goat IgG-FITC antibody at 37 °C for 4 h. Autophagosomes are shown in green puncta; the field magnification of induced autophagosomes are showed in a large square. (**B**) Cells unstimulated (Uns) or stimulated with ursolic acid (10 µg/mL) or oleanolic acid (20 µg/mL) for 48 h were fixed with 2.5% glutaraldehyde and post fixed with 1% osmium tetraoxide. Subsequently, the cells were dehydrated with ethanol and embedded in resin, and sections of 50 nm were obtained with an ultramicrotome. The field magnifications are shown in large squares and double membrane vesicles (autophagosomes) are shown with dark arrows. (**C**) Cells unstimulated (Uns) or stimulated with 10 µg/mL of ursolic acid or 20 µg/mL oleanolic acid for 48 h were fixed with 4% paraformaldehyde and washed with PBS, and subsequently the cells were incubated with anti-LC3 A/B and anti-LAMP1 at 4 °C overnight and then with anti-goat IgG-FITC and anti-mouse IgG-Rhodamine at 37 °C for 4 h. Autolysosomes are shown with white arrows. (**D**) The quantitative colocalization analysis of LAMP-1 with LC3-II signals (M/LAMP-1) and LC3-II with LAMP-1 signals (M/LC3-II) was performed with ImageJ Fuji to determine Mander’s coefficients from 0 to 1 (0 = non-overlapping images and 1 = colocalized images). No significant difference was found when both acids were compared. (**E**) Representative images of western blot for LC3 and quantitative analysis of LC3-II/LC3-I ratio. In some experiments 5 mM wortmannin (WORT) was added.

In order to describe whether ursolic and oleanolic acids stimulate the autophagy in A549 cells, monolayered cells were stimulated with triterpenes for 2, 6, 24, and 48 h. The immunofluorescence analysis for LC3-II (an autophagosome marker) demonstrated the null expression of this protein at 2 and 6 h after stimulus (data not showed). The expression of LC3-II in the cytoplasm of the cells was evident at 24 and 48 h after treatment (Figure 2A). As the LC3-II presence was more evident at 48 h after the stimulus, further experiments were undertaken at this time. The TEM analysis pointed out the presence of double-membrane vesicles and vesicles with degraded material in cells stimulated with ursolic or oleanolic acids (Figure 2B). To evaluate whether ursolic or oleanolic acid stimulates the complete autophagy flux, the samples were stained with anti-LC3-II and anti-LAMP1 (lysosome marker); the results revealed the colocalization of LC3-positive puncta with LAMP-1 (Figure 2C) and the colocalization coefficients indicated that both acids induced the formation of autolysosomes at 48 h. Both acids induce autophagy in A549 cells, and promote the complete autophagosomal maturation with subsequent autolysosome formation. To confirm the autophagy induction by triterpenes, western blot analysis was conducted. The LC3-II expression was increased in the case of stimulated cells and it indicates that autophagy was in progress. Densitometry analysis of LC3 demonstrated that ursolic acid augmented the ratio of LC3-II/LC3-I more than oleanolic acid. When wortmannin, a known inhibitor of autophagy, was added, the autophagy induced by triterpenes was reduced (Figure 2E).

### 2.3. Ursolic and Oleanolic Acids Induce Mitochondrial Changes in A549 Human Lung Cancer Cells

**Figure 3:**
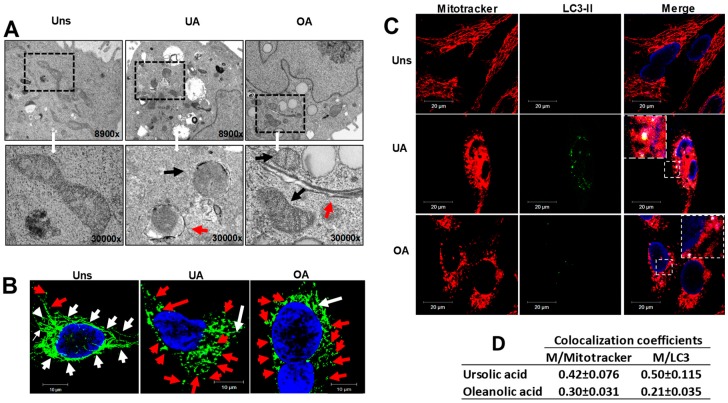
Ursolic and oleanolic acids promote mitochondrial morphology changes and induce mitophagy in A549 human lung cancer cells. (**A**) Cells unstimulated (Uns) or stimulated with 10 µg/mL ursolic acid (UA) or with 20 µg/mL oleanolic acid (OA) for 48 h were fixed with 2.5% glutaraldehyde and post fixed with 1% osmium tetraoxide. Subsequently, the cells were dehydrated with ethanol and embedded in resin, and sections of 50 nm were obtained with an ultramicrotome. The field magnifications are shown in large squares. Black arrows show the swollen mitochondria, with loss of cristae structure and increased mitochondrial lumen, while red arrows show mitochondria inside or near of double-membrane vesicles. (**B**) After treatment, the cells were incubated with MitoTracker Green (200 nM). White arrows show fused mitochondria; red arrows show fissioned mitochondria. (**C**) The treated cells were stained with MitoTracker Red (200 nM), fixed with paraformaldehyde 4%, washed with PBS and incubated with anti-LC3 A/B antibody at 4 °C overnight, and subsequently incubated with secondary antibody at 37 °C for 4 h. The large squares show a magnification of the area indicated in the small square and colocalization of the fragmented mitochondria and autophagosomes is shown. (**D**) The quantitative colocalization analysis of Mitotracker with LC3-II signals (M/Mitotracker) and LC3-II with Mitotracker signals (M/LC3-II) was performed with ImageJ Fuji to determine Mander´s coefficients from 0 to 1 (0 = non-overlapping images and 1 = colocalized images).

As it is well-known autophagy could be selective and mitochondria could be a target by autophagy. For that, the next step in this study was to examine whether ursolic and oleanolic acids induce ultrastructural alterations of the mitochondria. To this, TEM observations were analyzed. The ultrastructural analysis demonstrated that mitochondria of the treated cells showed several alterations, the cristae structure was modified and numerous mitochondria were swelled exhibiting a separation between the inner and outer membranes. Double membrane vacuoles containing mitochondria or formed around mitochondria were also observed (Figure 3A); suggesting that the autophagosomes formation was in progress. Next, we analyzed whether ursolic and oleanolic acids induce changes of the mitochondrial dynamics. Unstimulated or stimulated cells were stained with Mitotracker green. In unstimulated cells, mitochondria were found fused and forming a mitochondrial net (Figure 3B). In contrast, in the cells stimulated with ursolic or oleanolic acids, mitochondria were mostly fragmented and distributed in the cytoplasm (Figure 3B). To corroborate the presence of mitochondria sequestered by autophagosomes, untreated and treated cells were stained with anti-LC3-II and Mitotracker green and analyzed by confocal microscopy. Unstimulated cells showed a mitochondrial net without merging with LC3-II protein. In contrast, stimulated cells presented fragmented mitochondria, which colocalized with LC3-II (Figure 3C). The colocalization coefficients showed that in ursolic acid-treated cells a larger number of fragmented mitochondria were contained in autophagosomes, suggesting mitophagy. With oleanolic acid, the fragmented mitochondria sequestered by autophagosomes were minor (Figure 3D).

### 2.4. Mitophagy Induced by Ursolic and Oleanolic Acids is Associated with P62 Expression

**Figure 4:**
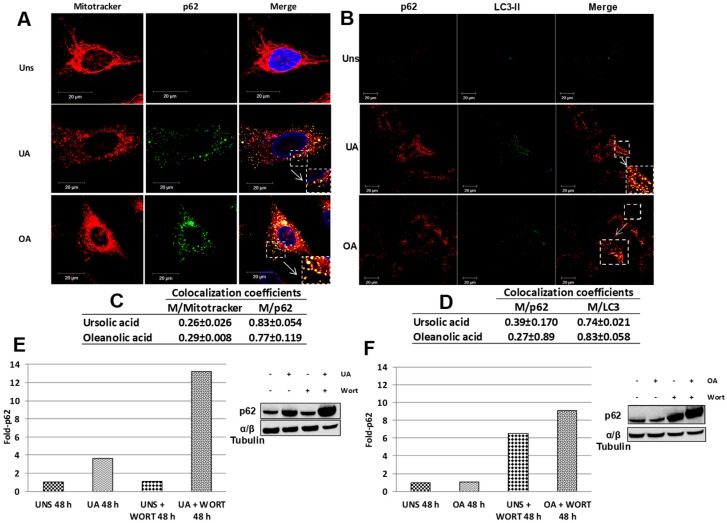
p62 colocalizes with fragmented mitochondria and LC3-II. (**A**) A549 cells unstimulated (Uns) or stimulated with 10 µg/mL of ursolic acid (UA) or 20 µg/mL of oleanolic (OA) for 48 h. The cells were stained with MitoTracker Green (200 nM), fixed with 4% paraformaldehyde and washed with PBS. The cells were incubated with anti-p62 at 4 °C overnight and finally incubated with secondary antibody at 37 °C for 4 h. The framed areas show the colocalization of p62 with fragmented mitochondria, and a magnification of these areas is displayed in the large squares. (**B**) A549 cells stimulated with 10 µg/mL of ursolic acid (UA) or 20 µg/mL of oleanolic (OA) for 48 h were fixed with 4% paraformaldehyde and washed PBS, and subsequently incubated with anti-LC3 A/B and anti-p62 at 4 °C overnight. Finally, the cells were incubated with secondary antibodies at 37 °C for 4 h. Squares and white arrows show the colocalization of p62 and LC3-II. (**C**) The Mander´s coefficients from 0 to 1 (0 = non-overlapping images and 1 = colocalized images) were determined, and the colocalization of Mitotracker with p62 (M/Mitotracker) and p62 with Mitotracker (M/p62) were calculated. (**D**) The colocalization degree of p62 with LC3-II (M/p62) and LC3-II with p62 (M/LC3-II) was determined through Mander´s coefficients from 0 to 1. (**E**,**F**) The plots showed the western blot analysis to detect p62 protein induced by ursolic or oleanolic acid in A549 cell without or with 5 mM wortmannin (WORT). The quantitative analysis of a representative experiment is shown.

Mitophagy is a type of selective autophagy that implies the capture and degradation of mitochondria. Some adaptor proteins are required for mitophagy induction as p62, also called sequestosome1 (SQSTM1). Thus, we evaluated the presence of p62 in unstimulated or stimulated cells. We observed that stimulus with ursolic or oleanolic acids induced the overexpression of p62 and this protein was found uniformly distributed in the cytoplasm (Figure 4A). Also, we observed the p62 protein colocalized with fragmented mitochondria (Figure 4A). Notably, for both acids, the colocalization coefficients were similar (Figure 4C). p62 is an adaptor protein that interacts with target proteins and with the LC3-II/GABARAP protein [11]; therefore, we performed immunofluorescence analyses against p62 and LC3-II. Both proteins were more expressed on stimulated cells (Figure 4B), and the colocalization coefficients between LC3 and p62 were high and similar for both acids, demonstrating that autophagosomes can be associated with p62 in this cell model (Figure 4D). However, a large number of p62 puncta seems to be independent on LC3-positive structures. Interestingly, the western blot analysis revealed that ursolic acid increases the fold-p62 (Figure 4E), which were overexpressed when autophagy was inhibited by treatment with wortmannin. In contrast, oleanolic acid just induces the p62 overexpression when autophagy was blocked (Figure 4F).

### 2.5. Triterpenes Trigger Different Signaling Pathways to Activate Mitophagy

**Figure 5:**
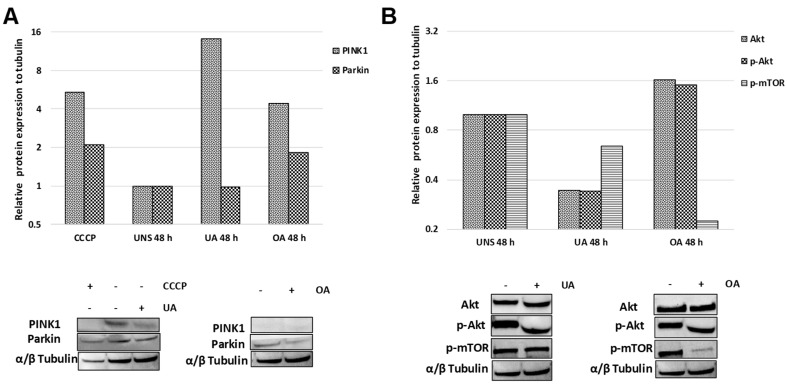
Ursolic and oleanolic acids trigger different signaling pathway to induce mitophagy. A549 cell monolayers were stimulated with ursolic acid (UA) or oleanolic acid (OA) during 48 h and western blot analyses were conducted. (**A**) The relative protein expression of PINK1 and Parkin in stimulated cells are shown. (**B**) The relative protein expression of Akt, p-Akt, and p-mTOR in stimulated cells are shown. The plots show the results of densitometry analyses of representative blots. CCCP was used as positive control of PINK1 and Parkin expression. Both acids were simultaneously analyzed in the same blots.

PINK1/Parkin axis is considered the key regulator of mitophagy, therefore, after cells were treated with ursolic or oleanolic acid, western blot analyses were conducted to evidence PINK1 and Parkin proteins. The results showed an important increase of PINK1 expression after ursolic or oleanolic treatment, suggesting the recruitment of PINK1 to outer membrane mitochondrial, however just an increase of Parkin in cells stimulated with oleanolic acid was observed. The treatment with ursolic acid did not modify the expression of Parkin (Figure 5A).

To evidence if AKT/mTOR pathway is involved in mitophagy induced by ursolic and oleanolic acid, the expression of AKT/p-AKT and p-mTOR were analyzed by western blot. Ursolic acid dramatically inhibited the expression of p-AKT and p-mTOR indicating that ursolic acid downregulate the AKT/mTOR pathway to turn on autophagy. Contrary, the treatment of oleanolic acid promoted a decrease of p-mTOR independent on p-AKT, suggesting the induction of autophagy induced by oleanolic acid is dependent on other signaling pathway (Figure 5B).

### 2.6. Ursolic and Oleanolic Acids Induce ROS Production and Nrf2 Activation

**Figure 6:**
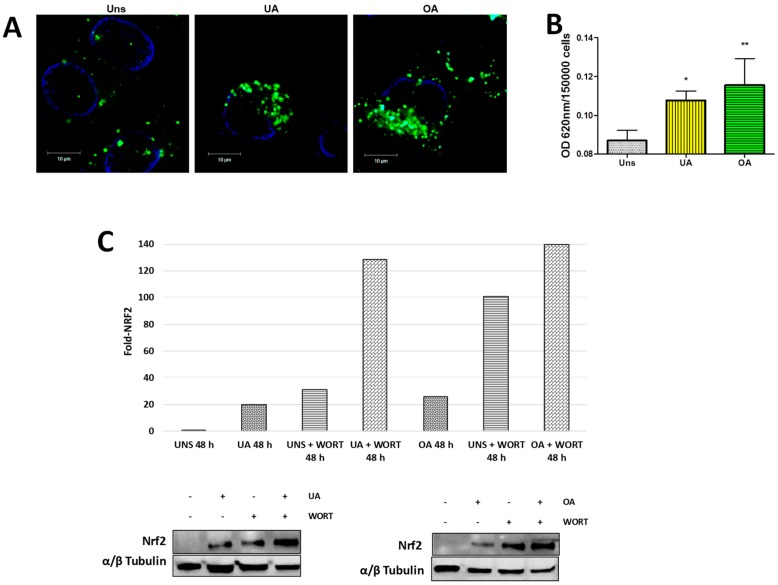
Ursolic and oleanolic acids induce ROS production and Nrf2 activation in A549 human lung cancer cells. (**A**) A549 cells unstimulated (Uns) and stimulated with 10 µg/mL ursolic acid (UA) or with 20 µg/mL oleanolic acid (OA) for 48 h were incubated with 2 µM DCFDA for 30 min. Subsequently, the cells were washed, fixed and analyzed by confocal microscopy. (**B**) ROS production was quantified with NBT reduction. NBT [80 µg/mL] was added to A549 cells and incubated for 15 min, and the formazan precipitates were diluted with working solution (2 M KOH + DMSO). The absorbance was analyzed by using an ELISA spectrophotometer at 620 nm. Error bars indicate the SD generated from three independent experiments, * *p* < 0.05 ** *p* < 0.01 One-way ANOVA with Dunnett’s post-test. (**C**) Nrf2 expression of A549 cells stimulated by ursolic and oleanolic acid without or with 5 mM wortmannin (WORT) was analyzed by Western blot. The plots show the densitometry analysis normalized with α/β-tubulin.

Mitophagy can be induced by ROS production, which leads to mitochondria membrane potential loss [26]. Thus, we evaluated whether ursolic or oleanolic acids induce ROS production in A549 cells. The stimulated cells showed a higher production of ROS than the unstimulated cells (Figure 6A). ROS production was quantified by NBT reduction assay. Statistically, both acids induced significant ROS production compared to unstimulated cells (Figure 6B). The high ROS production can be lead to oxidative stress-related damage and therefore to cell death. In order to avoid the damage provoked by ROS, cells have different molecular mechanisms antioxidants, one of them results in the activation of the nuclear factor erythroid 2-related factor 2 (Nrf2)/antioxidant response elements (ARE) signaling pathway. Experimental results showed an increase of Nrf2 expression after ursolic and oleanolic treatments. Remarkably, the inhibition of autophagy induced by triterpenes provokes a relevant increase of Nrf2 level (Figure 6C).

### 2.7. Autophagy Acts as A Pro Survival Mechanism against Ursolic and Oleanolic Acids Stimulus

**Figure 7:**
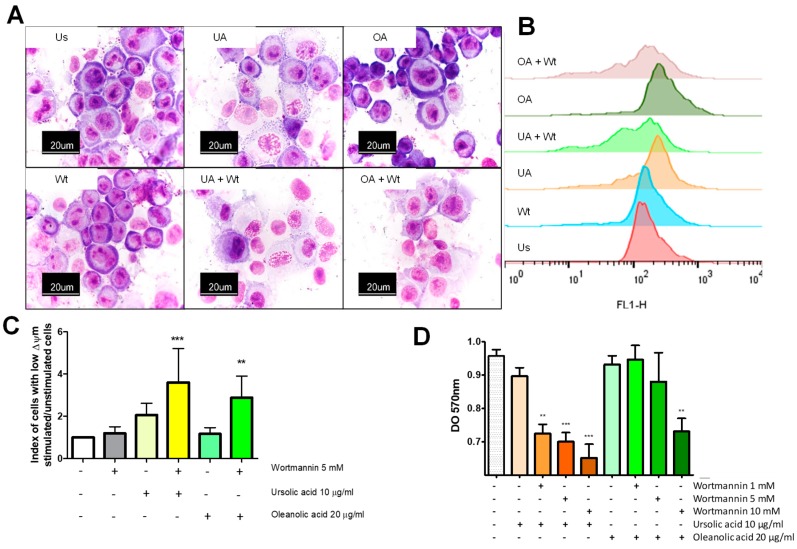
Autophagy inhibition induces morphological changes, low mitochondrial membrane potential (Δψ), and cellular death in A549 cells. (**A**) A549 cells unstimulated (Us) or stimulated with 10 µg/mL of ursolic acid (UA) or 20 µg/mL of oleanolic acid (OA) and 5 mM wortmannin (Wt) for 48 h were stained with Giemsa for 20 min. (**B**) The cells were stained with 1 mg/mL rhodamine 123 for 20 min and a representative histogram of the cells stained with rhodamine 123 is shown. (**C**) Index of cells with low mitochondrial membrane potential (Δψm). The data show the increased number of cells with low (Δψm) after treatment with ursolic or oleanolic acids plus wortmannin. Error bars indicate the SD from four experiments. (**D**) Treated cells were incubated with 1 mg/mL MTT for 30 min and precipitated formazan was dissolved with DMSO. The absorbance was measured by using an ELISA spectrophotometer at 570 nm. Error bars show the SD from six replicates of two independent experiments (**D**). ** *p* < 0.01 *** *p* < 0.001 One-way ANOVA with Dunnett’s post-test.

To establish the potential protective role of autophagy on the effect of the ursolic and oleanolic acids, we evaluated its participation in A549 cells. To this end, unstimulated cells and ursolic or oleanolic acid-stimulated cells were treated with wortmannin (an autophagy inhibitor) and stained with Giemsa. Wortmannin and oleanolic acid did not negatively affect the cellular morphology, as the cells appeared similar to the untreated monolayer. However, ursolic acid treatment induced changes in the cell nucleus, as decondensation and disorganization of chromatin were evident, and these changes were deeper in cells stimulated with ursolic or oleanolic acids plus wortmannin. Additionally, the cytoplasm in stimulated cells retained less dye than that in unstimulated cells (Figure 7A). Decreased mitochondrial membrane potential (Δψ) is associated with cellular stress, and if this stress continues, it leads to cellular death. Unstimulated and stimulated cells were stained with rhodamine 123 and analyzed in a cytometer. Cells treated with only wortmannin or stimulated with only oleanolic acid showed mitochondrial membrane potential (Δψ) similar to unstimulated cells, while cells stimulated with ursolic acid had a small population of cells with low mitochondrial Δψ, although this effect was not statistically significant. The index of cells with low mitochondrial Δψ significantly increases when cells were treated with ursolic or oleanolic acid plus wortmannin (Figure 7B,C). To corroborate the effect of wortmannin on the viability of the cells stimulated with ursolic or oleanolic acid, the reduction of MTT technique was assessed. The results showed a significant decrease in the metabolic activity of the cells treated with ursolic and oleanolic acid when they were incubated with wortmannin. This effect was stronger in cells treated with ursolic acid, which, a range of wortmannin concentration (1–10 mM) causes a significant decrease of metabolic activity; contrary, only 10 mM wortmannin was significantly different in the cells treated with oleanolic acid (Figure 7D).

## 3. Discussion

In cancer, the autophagy is a key cellular mechanism exhibited as a tumor suppressor pathway, but paradoxically, in some conditions autophagy can also promote the survival of cancer cells [27]. With this motivation, this work has been devoted to further identify particular conditions where autophagy presents advantages that may be useful in cancer applications. We study if the treatment of ursolic or oleanolic acids induces the autophagy in A549 cells as a protective response to ensure the survival cell. We demonstrated that the treatment of A549 cells with ursolic or oleanolic acid induced the expression of LC3-II and the formation of autophagosomes and autolysosomes (Figure 2). The results evidenced many changes of the mitochondrial ultrastructural morphology and the loss of the mitochondrial net. Moreover MET and confocal analysis demonstrated that under stimuli with ursolic or oleanolic acid, many mitochondria were enclosed in vacuoles with double membranes and colocalized with LC3-II (Figure 3). These findings pointed out that mitochondria are organelles targeted for autophagy, besides they correspond to the signature of a selective process, known as mitophagy. The clearance of damaged mitochondria by mitophagy has been reported as a mechanism involved in the resistance of chemotherapeutic-induced death [28]. Although some publications have reported the involvement of mitochondria in the biological properties of ursolic and oleanolic acid [29,30], to date, mitophagy has not been well established.

In mammalian cells the most typical pathway to mediate mitophagy is the PTEN-induced kinase 1 (PINK1)-Parkin signalling pathway [31]. However, recent studies have indicated the Parkin/PINK1 pathway is not responsible for all the mitophagy processes; in consequence, the mitophagy could be functional in the absence of Parkin [32]. Briefly, the loss of mitochondrial membrane potential and mitochondrial fragmentation induces the PINK1 kinase stabilization at the outer membrane mitochondrial and PINK1 recruit and phosphorylates Parkin directly [33]. Our results show that after mitochondrial net fragmentation, the expression of PINK1 was increased in A549 cells treated with ursolic or oleanolic acid, but, just the oleanolic acid slightly increases the Parkin protein level, indicating that the mitophagy process observed was independent on the activation of Parkin. Although the PINK1/Parkin pathway has been reported as a mediator of mitophagy in A549 cells stimulated by paraquat [34] and several isoforms of Parkin have been detected in A549 cell [35], our results suggest that ursolic or oleanolic acids induce a mitophagy process independent on Parkin in epithelial cells. These results comprise the first report that points out the induction of mitophagy by oleanolic and ursolic acids through the Parkin independent pathway. However, further studies are required to clarify the participation of others master mitophagy regulators, such as BNIP3 and NIX reported in tumorigenesis [36].

It has been previously reported that ursolic and oleanolic acids exert their anti-cancer properties through the downregulation of PI3K/AKT [37,38]. The PI3K/AKT/mTOR pathway is a relevant pathway that regulates intracellular signaling in different biological process, as apoptosis, differentiation, cell proliferation, and autophagy; additionally, it has been implicated in tumor growth and metastasis [39]. In the present study, we demonstrated for first time that autophagy induced in A549 cells after the treatment of ursolic acid was dependent on the inactivation of AKT/mTOR. The autophagy induction mediated by PI3K/AKT by ursolic acid has been reported in models of breast cancer cells and it has been associated with the autophagy protective in PC3 cells [4,37]. In contrast, in our model, the oleanolic acid diminished the p-mTOR expression trigger the autophagy; however, that autophagy is independent on AKT. The oleanolic acid not downregulate the p-AKT expression suggesting it induces a different pathway to stimulate the autophagy process. Our results are in good agreement with Liu and colleagues who described that the activation of JNK and inhibition of mTOR lead to autophagy in PANC-1 and A549 cancer cells [17].

The removal of mitochondria by selective autophagy comprises the involvement of adapter proteins that acts as a bridge between the mitochondria and LC3-II protein to form the autophagosome. Several proteins have been described as adapter proteins, p62, NBR, and optineurin between others [11]. Here, we particularly analyzed the role of p62 protein after oleanolic or ursolic treatment, we observed a high p62 expression, which colocalized with mitochondria and LC3-II (Figure 4). Additionally, the p62 protein was overexpressed in our model when autophagy was blocked with wortmannin, suggesting autophagy is necessary to automatically regulate the expression of p62 stimulated by triterpenes. Moreover, although the increase of p62 in carcinoma cell has been established [40], it is possible hypothesize that the large number of p62 structures induced by inhibition of autophagy in our model is due to that p62 colocalize upstream autophagy factors such as ULK1 and VMP1 in the autophagosome formation sites; as it has proposed Itakura and Mizushima [41].

On the other hand, some studies have shown that oleanolic acid induced ROS accumulation, which contributed to autophagy cell death in HepG2 cells [7], and the ROS induced by ursolic acid has been implicated in the autophagy of U87MG cells [18]. We observed the increased production of ROS induced by treatment with ursolic or oleanolic acid. The high production of ROS may provoke oxidative damage, affects mitochondrial potential and lead to cell death [42,43]. Although, the role of ROS in mitophagy remains unclear, a recent report suggested that ROS may act as a trigger for the induction of PINK1/Parkin-dependent mitophagy [43]. Both ursolic and oleanolic acids have been demonstrated to alter mitochondrial membrane potential [22,23,24,25] but we only observed this effect with ursolic acid treatment. In our model, we inhibited the autophagy to evaluate if this process represents a valuable contribution pro-survival of the cancer cells. We found the wortmannin caused a significant increase of the index of cells with low mitochondrial Δψ and a decrease of metabolic activity, indicating the damage of the cells (Figure 7), and suggesting that the autophagy has an important role as a biological mechanism regulator of survival of these cells. As mentioned above, when we inhibited the autophagy with wortmannin, p62 was over-expressed, and taking into account that the p62 protein level increases after oxygen radical stress [44], consequently the cellular response against highest ROS production can be related to p62. The p62 promoter contains an antioxidant response element (ARE) and is up-regulated by oxidative stress via Nrf2 [45]. Thereby, the increase of p62 protein could be related to the activation and signaling of Nrf2. Nrf2 is a transcription factor responsible for gene expression of a series of anti-oxidant proteins and detoxifying enzymes [46]. Also, the activation of Nrf2 depends on PI3K/AKT signaling pathway [47]. We found an over expression of Nrf2 when PI3K was inhibited with wortmannin, this increase could be a redundant effect caused by p62 protein, which also was increased by wortmannin. The activation of p62 and Nrf2 indicates that these proteins could be chemically responsible for ROS detoxification promoting the survival of the cells in autophagy privation. Comparative experiments related to the effect of ursolic and oleanolic on normal cells have previously described changes in chemical activity in respect to cancer cells [48]. It has been noted that oleanolic acid can be responsible for an induced autophagy in normal cells mediated by inactivation of Akt/mTOR/S6K signaling [49]. Moreover, ursolic acid can stimulate the autophagy through particular PI3K/Akt/mTOR signaling pathways [50]. Those reports clearly indicate that triterpenes are able to induce autophagy as a homeostatic mechanism. All these results together to our findings can be considered for pointing out that autophagy and p62 could be important therapeutic targets for inhibition in cancer.

## 4. Materials and Methods

### 4.1. Reagents

A549 cells (ATTC CCL-185) were purchased from American Type Culture Collection (ATCC, Manassas, VA, USA). F12 culture medium (21700-018), penicillin-streptomycin (15140-122), and fetal bovine serum (12483-020) were purchased from Gibco Thermo Fisher Scientific (Waltham, MA, USA). The triterpenes used in all experiments were obtained from SIGMA (St. Louis, MO, USA) (+)-Ursolic (U6753) and (+)-Oleanolic (O5504) acids, for convenience they were handled in the text as ursolic acid (UA) or oleanolic acid (OA). Thiazolyl blue tetrazolium bromide (M5655), nitrotetrazolium blue chloride (N6876), Carbonyl cyanide 3-chlorophenylhydrazone, CCCP (C2759), 2′,7′-dichlorofluorescein diacetate (D6883), Hanks balanced salt solution (H1387), wortmannin (W1628) and rhodamine 123 (R8004) were purchased from Sigma (St. Louis, MO, USA). Anti-LAMP1 (SC-20011, clone H4A3), anti-LC3 A/B (SC-16756 clone F-14), anti-goat IgG-TRITC (SC-2490), anti-Nrf2 (SC-722), anti-goat IgG-FITC (SC-2024), anti-rabbit IgG-TRITC (SC-2367), and RIPA Lysis Buffer System were obtained from Santa Cruz Biotechnology (Dallas, TX, USA), and anti-p62 (ab91526) was purchased from Abcam (Cambridge, UK). Mitotracker Green FM (M7514) was obtained from Invitrogen (Carlsbad, CA, USA). Protein assay dye (5000006), Stain-Free gels 4–20%, and polyvinylidene fluoride membrane (PVDF, 162-0115) were purchased from Biorad (Hercules, CA, USA). Anti-LC3 A/B (C02-4108S), Phospho-mTOR (Ser2448) (2448), Anti-PINK1 (D8G3) (6946), Anti-Parkin (Prk8) (4211), and Anti-α/β tubulin (C02-2148S) were purchased from Cell Signaling Technology, Inc. (Danvers, MA, USA). Glutaraldehyde (16200), osmium tetroxide (19100), propylene oxide (20401) and epoxy resin (14660) were purchased from Electron Microscopy Sciences (Hatfield, PA, USA).

### 4.2. Cell Culture

A549 cells, a tumoral cell line derived from neumocyte type II epithelial cells, were grown in F12 culture medium supplemented with fetal bovine serum at 10%, penicillin at 100 units/mL, and streptomycin at 100 µg/mL and maintained at 37 °C in a humidified atmosphere comprising 95% O_2_ and 5% CO_2_. The cells were detached with 0.25% trypsin, counted in a Neubauer chamber and seeded according to each experiment. The next day, the cells were treated with ursolic acid or oleanolic acid, and wortmannin, in some experiments. After of stimuli, the cells were washed with Hanks balanced salt solution (HBSS) and processed according to each experiment.

### 4.3. Cell Viability Assay

A549 cells (1 × 10^4^ cells/well) were plated in a 96-well plate and incubate by 24 h at 37 °C. Then, cells were washed two times with HBSS and next were stimulated with 5, 10, 20, and 40 µg/mL oleanolic or ursolic acid. After 24 and 48 h of incubation, 0.4% trypan blue solution was added. The cells that excluded trypan blue were identified as viable cells. To calculate the viability percentage, a total of 4 random fields were quantified. According with these results, the next experiments were carried out with 10 µg/mL ursolic acid or 20 µg/mL oleanolic acid during 24 and/or 48 h.

### 4.4. Immunofluorescence

A total of 2 × 10^5^ A549 cells were seeded onto glass coverslips in 6-well plates and treated with ursolic or oleanolic acid for 2, 6, 24, or 48 h according to each experiment. After washing with HBSS and fixing with 4% paraformaldehyde at 4 °C for 20 min, the cells were permeabilized with a solution of 0.2% Triton X-100, and a solution of 4% bovine serum albumin (BSA) was used to block unspecific antibody interactions. The cells were incubated with primary antibodies (anti-LC3 A/B, anti-LAMP1 or anti-p62, according the experiment) at 4 °C overnight. Unbounded primary antibody was washed away with phosphate buffer solution (PBS) and secondary antibody was incubated at 37 °C for 4 h. The samples were analyzed by using a confocal system coupled to an invertide microscope (LSM5 PASCAL Zeiss, Jena, Germany). The quantitative colocalization analysis of two fluorescent signals was performed with ImageJ Fuji to determine Mander´s coefficients from 0 to 1 (0 non-overlapping images and 1 colocalized image).

### 4.5. Western Blotting

Cells grown on culture bottle were treated with ursolic or oleanolic acid during 24 and 48 h. After treatment, cells were detached with RIPA Lysis Buffer System and the total protein was quantified by the Bradford method. Equal amounts of protein (50 µg) were loaded into the wells of the Stain-Free gels (4–20%) and an SDS-PAGE was performed. Then, the proteins were transferred to a polyvinylidene fluoride membrane (PVDF). After, membranes were blocked with skimmed milk 5% in Tris Buffer Solution containing 0.05% Tween 20 (TBST) for 1 h. Then, the membranes were incubated overnight at 4 °C with primary antibodies: anti-p62, anti-PINK1, anti-Parkin, anti-Akt, anti-phospho-Akt, anti-phospho-mTOR, anti-Nrf2, or anti-LC3-II, according with instructions of the manufacturing company. Subsequently, the blots were washed three times with TBST, and next, the membranes were incubated with horseradish peroxidase-conjugated secondary antibody (1:5000) for 1 h. After washing, the bands were detected by using a chemiluminescence method and photographed by Chemidoc Touch, Image system (Biorad, Hercules, CA, USA). All bands were normalized with β-actin or α/β-tubulin as an internal control.

### 4.6. Ultrastructural Analysis

A total of 1 × 10^6^ cells were seeded onto Petri dishes. The cells unstimulated and stimulated for 48 h were washed with HBSS and fixed with a solution of 2.5% glutaraldehyde for 2 h at room temperature, and then, the cells were washed with PBS and detached with a cell scraper. The cells were post fixed with 1% osmium tetroxide for 2 h. Subsequently, the cells were dehydrated in graded ethanol at 70%, 80%, 96% and absolute alcohol, washed with propylene oxide, embedded in epoxy resin and sectioned on an ultramicrotome at 70–80 nm (Ultracut UCT, Leica Microsystems, Buffalo Grove IL, USA). The samples were contrasted by using uranyl acetate and lead citrate. Images were captured with a transmission electron microscope (Tecnai 10, Phillips, Amsterdam, NL, USA).

### 4.7. Staining with MitoTracker Green

A549 monolayers with 2 × 10^5^ cells were prepared on glass coverslips; the cells were stimulated for 48 h and washed with HBSS. Then, a solution of 200 nM MitoTracker green FM was added, and the cells were incubated at 37 °C for 30 min. Subsequently, the cells were washed with HBSS and fixed with 4% paraformaldehyde at 4 °C for 20 min. The samples were analyzed by using a confocal system coupled to an invertide microscope (LSM5 PASCAL Zeiss, Jena, Germany).

### 4.8. Reactive Oxygen Species Detection

To measure the ROS induced by oleanolic or ursolic acid, 5 × 10^4^ cells were seeded onto a 24-well plate. The cells unstimulated and stimulated for 48 h were incubated with a solution of 1 µg/mL NBT for 15 min, and the formazan crystals were subsequently dissolved with a 2 M DMSO-KOH solution. The absorbance was analyzed at wavelength of 620 nm in an ELISA Multiskan spectrophotometer (Thermo Fisher Scientific, Waltham, MA, USA). Additionally, the ROS were detected by immunofluorescence using 2 × 10^5^ cells seeded into coverslips in a 6-well plate. The cells untreated and treated for 48 h were washed with HBSS and incubated at 37 °C for 30 min with a solution of 2 mM DCFDA diluted in HBSS. Finally, the cells were washed and fixed with 4% paraformaldehyde. The samples were analyzed by using a confocal system coupled to an invertide microscope (LSM5 PASCAL Zeiss, Jena, Germany).

### 4.9. Staining with Rhodamine 123

To evaluate the mitochondrial changes, staining with rhodamine 123 was performed. Approximately 4 × 10^5^ cells were seeded onto a 6-well plate, and these cells were untreated or treated with triterpenes and/or 5 mM wortmannin for 48 h. Then, the cells were detached with trypsin and washed with PBS. Next, the cells were incubated with 1 µg/mL rhodamine 123 in the dark at room temperature for 20 min. Subsequently, the cells were washed twice with PBS, and 1 × 10^5^ cells were acquired in a FACSCalibur cytometer (BD, San Jose, California) and analyzed by using the CellQuest Pro software 6.1.

### 4.10. MTT Reduction

A total of 1 × 10^5^ cells were seeded onto a 24-well plate. The cells unstimulated and stimulated with 10 µg/mL ursolic acid, 20 µg/mL oleanolic acid and/or 1, 5, 10 mM wortmannin for 48 h were incubated with a 1 mg/mL MTT solution at 37 °C for 3 h. Subsequently, the MTT solution was removed, and 200 µL of DMSO was added to solubilize the formazan precipitates, and finally the absorbance was quantified at a wavelength of 570 nm in an ELISA Multiskan spectrophotometer (Thermo Fisher Scientific, Waltham, MA, USA).

### 4.11. Statistical Analysis

Statistical analysis was performed by using the GraphPad Prism 5.0 (San Diego, CA, USA) statistical software. Data represent the mean ± SD of two or three independent experiments. The data were analyzed with one-way or two-way ANOVA, with Dunnett’s post-test. The differences were considered to be statistically significant when * *p* < 0.05, ** *p* < 0.01, or *** *p* < 0.001.

## 5. Conclusions

The importance of this work comes from the clear indication about the chemically selective influence of typical natural acids that may take part in biological conditions for cancer treatments. In the present study, we demonstrated for first time that ursolic and olenolic acids induce mitophagy through the Parkin independent pathway in A549 human lung cancer cells. Also, we provide a better understanding about the pivotal role of autophagy/mitophagy and p62 protein as responsible for survival of the A549 cells treated with ursolic or oleanolic acids. Altogether our findings highlight the regulation of mitophagy and SQSTM1/p62 protein as therapeutic strategies in cancer.
